# Informal Caregivers of Patients with Disorders of Consciousness: a Qualitative Study of Communication Experiences and Information Needs with Physicians

**DOI:** 10.1007/s12152-022-09503-0

**Published:** 2022-07-23

**Authors:** Karoline Boegle, Marta Bassi, Angela Comanducci, Katja Kuehlmeyer, Philipp Oehl, Theresa Raiser, Martin Rosenfelder, Jaco Diego Sitt, Chiara Valota, Lina Willacker, Andreas Bender, Eva Grill

**Affiliations:** 1 Institute for Medical Information Processing, Biometry and Epidemiology, Ludwig-Maximilians-Universität München, Marchioninistraße 15, 81377 Munich, Germany; 2 Pettenkofer School of Public Health, Munich, Germany; 3 Department of Biomedical and Clinical Sciences ‘Luigi Sacco’, University of Milano, Milan, Italy; 4 IRCCS Fondazione Don Carlo Gnocchi, Milan, Italy; 5 Institute of Ethics, History and Theory of Medicine, Ludwig-Maximilians-Universität München, Munich, Germany; 6 Therapiezentrum Burgau, Hospital for Neurological Rehabilitation, Burgau, Germany; 7 Department of Neurology, University Hospital of the Ludwig-Maximilians-Universität München, Munich, Germany; 8 Institut du Cerveau et de la Moelle epiniere (ICM), Sorbonne Universités, Paris, France; 9 NeuroSpin Center, Institute of BioImaging Commissariat à l’Energie Atomique, Gif/Yvette, France; 10 German Center for Vertigo and Balance Disorders, Klinikum der Universität München, Munich, Germany

**Keywords:** Disorder of consciousness, Caregivers, Information needs, Effective communication, Functional Neurodiagnostics, Qualitative study

## Abstract

Due to improvements in medicine, the figures of patients with disorders of consciousness (DoC) are increasing. Diagnostics of DoC and prognostication of rehabilitation outcome is challenging but necessary to evaluate recovery potential and to decide on treatment options. Such decisions should be made by doctors and patients’ surrogates based on medico-ethical principles. Meeting information needs and communicating effectively with caregivers as the patients´ most common surrogate-decision makers is crucial, and challenging when novel tech-nologies are introduced. This qualitative study aims to explore information needs of informal DoC caregivers, how they manage the obtained information and their perceptions and experiences with caregiver-physician communication in facilities that implemented innovative neurodiagnostics studies. In 2021, we conducted semi-structured interviews with nine caregivers of clinically stable DoC patients in two rehabilitation centers in Italy and Germany. Participants were selected based on consecutive purposeful sampling. Caregivers were recruited at the facilities after written informed consent. All interviews were recorded, transcribed verbatim and translated. For analysis, we used reflexive thematic analysis according to Braun & Clarke (2006). Caregivers experienced the conversations emotionally, generally based on the value of the information provided. They reported to seek positive information, comfort and empathy with-in the communication of results of examinations. They needed detailed information to gain a deep understanding and a clear picture of their loved-one’s condition. The results suggest a mismatch between the perspectives of caregivers and the perspectives of medical profession-als, and stress the need for more elaborate approaches to the communication of results of neu-rodiagnostics studies.

**Supplementary Information:**

The online version contains supplementary material available at 10.1007/s12152-022-09503-0.

## Introduction

Disorders of Consciousness (DoC) may be the result of severe traumatic or non-traumatic brain injuries leading to a state of coma. In this state, patients are typically not capable of opening their eyes even on rather rigorous stimulation nor show any behavior that suggests the presence of “psychologically interpretable contact with the outside world “[1]. From the coma state, some patients transit into a state of wakefulness, in which they open their eyes intermittently without any evidence for an ability to make contact or of awareness [1]. This state is called the unresponsive wakefulness syndrome (UWS) [2]. A patient is considered to be in a minimally conscious state (MCS) as soon as there is any evidence for cognitive function and reproducible behavior (eg. following commands or answering simple questions verbally or gesturally), which suggest awareness of the environment [1, 3]. Patients emerge from MCS when they are capable of communication. Within the broader diagnostic category, clinicians distinguish between the subcategories MCS- and MCS+. These categories are diagnosed correspondent to the presence (MCS+) or absence (MCS-) of behavioral evidence of receptive or expressive language function [4]. DoC differs from the cognitive motor dissociation (CMD), a clinical entity which has been newly introduced within the past years to describe the phenomenon of detected conscious awareness while behavioral examination cannot but diagnose absent or low-level responsiveness [5]. Coma, UWS, MCS and CMD are termed DoC.

Due to continual improvements in rescue and emergency services and intensive care management, the number of patients with UWS in Europe is increasing, ranging now from 0.2/100000 to 6.1/100000 inhabitants [6–10]. In Germany, an estimated 1500–5000 persons are now living with an UWS [10]. In Italy, an estimated of 1500 patients suffer from UWS [11]. Informal caregivers play a vital role in the treatment process of patients with DoC since in most of the cases they become the surrogate health decision-makers, with the task to express the predermined or presumed will of the patients.

However, evidence-based diagnostic criteria and the ability to assess states of consciousness and cognitive function are yet limited. In clinical practice, it is still challenging to diagnose DoC accurately and to distinguish UWS from MCS. This becomes apparent with rates as high as 43% of misdiagnoses of UWS when in fact patients show some kind of consciousness [12]. One major issue in the diagnosis of MCS is the inconsistency of behaviors demonstrating consciousness within examinations. In many patients, the state of consciousness is not stable but fluctuates enormously even over brief periods of time [13]. However, for consciousness to be determined, it is necessary that the meaningful response to a stimuli that is shown is reproducible [3].

Tailored and individualized neurodiagnostic pathways are determined by the patients´ behavior, EEG-based techniques and neuroimaging [14–18]. The state of the art of a thorough diagnosis of DoC is based on standardized clinical examinations, using validated rating scales, such as the Coma Recovery Scale – Revised (CSR-R) [19]. Modern, technology-based methods, namely functional neurodiagnostics, can provide additional information and increase the certainty of diagnosis. For example, high density EEG (HD-EEG), functional MRI (fMRI), and the combination of repetitive transcranial magnetic stimulation and HD-EEG (rTMS/HD-EEG) can detect signatures of consciousness without the need for behavioral command following [20]. These methods can be enhanced through analyses with machine learning algorithms and other artificial intelligence methods and have already shown promising results [1, 20].

Despite the diagnostic advances being made, the remaining diagnostic as well as prognostic uncertainty poses a great challenge to physicians, especially when it comes to communicating test results to DoC caregivers. It is a great challenge for medical professionals and even more so for lay caregivers to grasp their meaning and consequences (recovery potential, pain management, quality of life, to name a few), because they are very complex in its novelty. Yet, accurate diagnostic results are important clinically as well as morally as they inform medical decisions and influence clinical outcomes as well as give arguments for the justification of rehabilitation measures [21]. Pan et al. (2020) has shown that diagnostic accuracy correlates with consciousness recovery [22]. Diagnosis of DoC and prognostication of its outcome may be challenging although necessary to evaluate recovery potential and to decide on further treatment strategies.

Accurate diagnoses have not only implications for the decisions about the patients´ care but also affect caregivers in their emotional coping with the uncertainty of the situation and their responsibility to participate in the shared clinical decision-making process. It is necessary for caregivers to gain an understanding for what the test results indicate and what their consequences might be. However, the complex nature of functional neurodiagnostics can be overwhelming for informal caregivers and their results can be hard to grasp, let alone their consequences.

Informal caregivers of patients with DoC are not detached rational agents, they face high levels of distress taking care of their family member. While being exposed to an emotionally complex and life changing situation, they are confronted with health-related decisions for the DoC patient. Several studies report low mental and physical health, and high levels of distress caused by the ambiguity of the new situation, the uncertainty of its duration, the possible suffering of the patient and of its final outcome [8, 23–25]. Targeted communication strategies are necessary to support DoC caregiver’s coping with this complex and difficult situation and at the same time surrogate decision-makers have to understand the patient’s prognosis as a basis for their participation in the clinical decision-making process.

To find ways to address caregivers appropriately in their difficult double role, it is crucial to investigate needs and demands of DoC patient’s informal caregivers. It has been shown that a large proportion have a high need for information and communication, while expressing high emotional burden [8]. Specifically, information about prognosis, therapy effects and alternatives to treatment continuation seem to be essential [26, 27].

In dedicated acute rehabilitation facilities, communication of the diagnosis and prognosis of recovery potential is a crucial moment to pave the way for a curative or palliative treatment strategy. Caregivers’ communication needs should be the basis of any communication strategy to ensure that all involved parties base their actions and decisions on a shared understanding of the patient’s condition. At the same time, caregivers’ coping process should be monitored to make sure that they are not overwhelmed with the burden of surrogate decision making. While the available data show evidence of high psychological distress and emotional burden in caregivers, informational needs and communication preferences of DoC informal caregivers are still incompletely understood. Likewise, it is still unclear which information may be relevant to informal DoC caregivers and which may even cause unnecessary distress or confusion.

The aim of this study is to explore information needs of informal DoC caregivers, how they manage the obtained information and their perceptions of and experiences with caregiver-physician communication.

## Methods

We orient ourselves towards the COREQ-Checklist after Tong et al. [28] in the reporting of our study. The qualitative study presented here is part of the PerBrain project, which is set within the European ERA PerMed initiative. The project is registered under the Identifier NCT04798456 [29].

We chose to answer our research question within the qualitative paradigm as we were interested in exploring the participants´ experiences and views. The qualitative research approach is characterized by an inductive bottom-up process, which seeks to generate hypotheses and works with interpretative and descriptive analysis methods. Small sample sizes enable rich descriptions of individual cases [30]. We conducted semi-structured interviews and used the reflexive thematic analysis (TA) according to Braun and Clarke [31, 32] as the method for analysis.

### Research Team and Reflexivity

Interviews were conducted by two researcher assistants, PO and CV. PO, who conducted the German interviews (PO), is male. He holds a Bachelor degree in Psychology and is currently an undergraduate student and PhD candidate for a medical degree at the Ludwig-Maximilians-Universität in Munich. He had no experience in conducting qualitative interviews until he participated in an interview training prior to the data collection. Outside of the research setting, there was no point of contact between the study participants and the researcher.

The female interviewer for the Italian interviews (CV) is a clinical psychologist with an expertise in psychotherapy. She works at a neurological rehabilitation hospital as a clinical psychologist consulting caregivers of patients with DoC. Prior to conducting the interviews she had conducted psychoeducational counselling with some of the participating caregivers.

The female first author responsible for the analysis of the material (KB) is a professional nurse with a Bachelor degree in nursing science and a Master degree in Public Health at the Ludwig-Maximilians-Universität in Munich. Beforehand, she had gathered experience with qualitative research using grounded theory. Several co-authors supported the data collection (AC, PO, MR, TR, CV, LW). The other authors (AB, MB, EG, KK, JDS) are senior researchers who carry several years of experience within the research field. They designed, supervised and reviewed the author intermittently. EG was the PI of this sub-study.

### Theoretical Framework

A post-positivist critical realist approach underpins the methodological orientation and theory of this qualitative work. Critical realism is an epistemological position that is critical of the ability to know reality with certainty. This post-positivist position on the one hand seeks an objective truth and reality but at the same time acknowledges the fact that reality can only be known imperfectly. This position includes the assumption that all observations are theory-laden, biases are undesirable but inevitable and inherent to each researcher, resulting from one’s world views or cultural experiences [33, 34].

We argue that the post-positivist critical realist approach is well suited for our study because we aim to describe the study participant’s perceived reality as truthfully as possible and on the other hand acknowledge that our own reflexivity influences the interpretation of data.

### Participant Selection

This study was part of a larger project with a mixed methods approach. Functional neurodiagnostic test were applied to those patients who had been enrolled in the quantitative part of the study. Test results were disclosed to their caregivers who had consented to participate in the larger study. Physicians who disclosed the results, were aware of the study, knew that the caregiver and the respective patient participated in the quantitative caregiver study but did not know if the caregiver would consent to the qualitative interviews.

The following methods of neurodiagnostics were used: repeated application of the CRS-R (Italy and Germany), fMRI (Italy), structural MRI (Italy and Germany), HD-EEG with resting state and active paradigms (Italy and Germany), TMS/EEG (Italy), standard EEG (Italy and Germany), FDG-PET (Germany).

We used consecutive purposive and maximum variation sampling. Participants were recruited in two institutions in Germany and Italy. Physicians from two neurological rehabilitation facilities near Munich and Milan acted as gatekeepers. Potential participants were included into the study, when they acted as informal caregivers, eg. the person responsible in terms of time dedicated to care and health-related decision-making. The study participants had to be 18 years and older, emotionally stable and fit for the study, fluent in German and Italian, respectively, and had to have signed the informed consent form. Caregivers were included based on the patients´ characteristics: clinically stable patients hospitalized for at least three weeks, aged 18 years and older and suffering from a DoC (coma, UWS, MCS) as determined by repeated (5 times) CRS-R. We purposefully selected caregivers who had the potential to benefit considerably from functional neurodiagnostics, i.e. we selected participants for qualitative interviews in clinical cases where there was a potential discrepancy between the perceived clinical phenotype and the result of multimodal functional neurodiagnostic testing, eg. the patient was clinically in UWS, and functional diagnostic showed him to be in MCS, or the patient was clinically in UWS and the test results confirmed UWS. Also, we followed the principles of maximum variation to achieve heterogeneous responses. One to two weeks before the interviews were conducted, the participants had a comprehensive conversation with a neurologist for the disclosure of the functional neurodiagnostic test results.

### Setting

All but one of the German interviews took place in presence, in a private, well-lit examination room of the study site. A desk and two chairs were used to set up the interview setting according to qualitative interview process recommendations [35]. The interviewer and the participant sat over corner. Due to the ongoing Covid-19 pandemic, hygiene measures such as maintaining social distance and wearing a medical mask had to be applied.

One of the German interviews was conducted via telephone. All of the Italian interviews were conducted via video-calls for Covid-19 regulatory reasons. In all of the interviews, solely one interviewer (PO, CV) and one participant were present.

### Data Collection

Semi-structured interviews were conducted using an interview grid. It had been developed according to Helfferich [35]. The interview grid was structured into seven mandatory leading questions as an invitation to tell (Table 1), as well as optional concrete questions and guiding questions or questions to continue. The full interview grid can be seen in the Supplementary Information. It was pre-tested and reviewed. We did so by meeting in workshops to discuss our understanding of the questions and whether they were able to address our research question. The grid was adjusted according to each subgroup’s feedback. We then conducted a mock interview in each country-group to see how the grid worked in a more realistic setting. Both mock interviews were not included into the study because the team had a priori defined not to use them. The structure of the interview grid ensured that all relevant themes were covered in all of the interviews and that the conversation targeted towards the research interest. At the same time, it gave enough room for individual expression of themes that have been relevant to the participants.

**Table 1:** Leading questions for semi-structured interviews

**1**	We are interested in your experience with the last counselling (the last big conversation that you had with the doctor/medical team) where you were informed about the neurological examinations of X^a^. Please, tell me all you think is important in this regard. Simply start from what comes to your mind first.
**2**	In how far did that conversation differ from other experiences of medical communication?
**3**	What have you expected of the neurological examinations that were conducted here?
**4**	Could you tell me more about X’s specific neurological examinations?
**5**	How were the results of these neurological examinations communicated to you?
**6**	Could you describe how that affected how you think about the patient?
**7**	In how far could the delivery of neurological information about X be improved in your point of view?
**8**	Is there anything that is important to you, that we have not talked about enough during our interview?

^a^Name or role of the family member

The interviews were carried out by two researchers (PO, CV). To capture the interview atmosphere and immediate impressions, field notes were taken post-interview using a post-script-template (PO, CV). The audiotaped interviews were then transcribed verbatim (KB, CV) using the f4-transkription software (Dresing & Pehl GmbH, Marburg, Germany, http://www.audiotranskription.de/), following predefined transcription and anonymization rules. Transcripts were not returned to participants for correction. Afterwards, the interviews were translated into English (KB, MB, TR, LW). No repeat interviews were carried out. The sample size was determined by thematic saturation.

### Data Analysis

We chose the reflexive TA as a method to identify, analyze and report patterned meaning across a dataset [31]. The chosen method is widely accepted and has been used increasingly in social and health sciences over the past decade [36]. We chose this method because it is suitable to address our research question, including questions on experiences and views. We present a small and homogenous sample which meets the suggestions for sampling [32]. By using the TA as the method of analysis, we are able to develop conceptually sound interpretations of data [31].

The analysis was conducted by the first author of the study and the results were discussed with the co-authors in various stages of the analysis. It started parallel to the data acquisition. It followed the six steps of the reflexive TA within the social post-positivist critical realist paradigm, namely to familiarize with the data (1), generate initial codes (2), search (3), review (4), define and name the themes (5) and producing the report (6) [32]. The process of analysis was recursive and iterative. Codes and themes were developed on a latent level and inductively from the data material.

The familiarization (phase 1) with the data items began with the transcription, the reading of the material and taking notes on what seemed relevant in regard to the research question. Having done this, initial codes were generated systematically across the entire data set in phase 2. After this, KB searched for themes to collate codes and gather all data relevant to each potential theme (phase 3). Additionally, we constructed categories and sub-categories to give the themes more structure. To ensure theme coherence, phase 4 was about examining whether the themes fit in relation to the coded extracts and the entire data set. Whereas the analysis resulted in seven themes initially, it was downsized to three themes in the revision phase. Thereby the remaining themes became thicker and more distinctive from each other. In phase 5, we defined and named the themes to refine their specifics, and the overall story the analysis had developed. Finally, the process of analysis was closed by writing the report and answering the research question (phase 6). Constant reflection of our own positions regarding our subjective understandings and presuppositions were an integral part of our reflexive analysis of the participants´ experiences.

For technical implementation of the coding and analysis, the MAXQDA-Software 20.4.1 (VERBI Software; Consult, Sozialforschung GmbH, Berlin, Germany) was used.

### Data Protection and Ethics

Prior to commencing the study, approval from the Ethics Committee and the Data Protection Officer of the Medical Faculty of the Ludwig-Maximilians-Universität in Munich was obtained (Date:28.08.2020/No.:20–634)), as well as from IRCCS Fondazione Don Carlo Gnocchi in Milan (No.: 08_25/07/2019). The interviewers were especially qualified to adequately react to signs of burden within the interviews. Participation was voluntary and informed consent forms were signed after the participants were informed about the purpose of the study, the anonymization process of the gathered data, data protection measures and the confidentiality of the interviews. At any time, participants had the opportunity to withdraw consent. The pseudonymized data will be stored in a protected environment for 10 years after the final publication of the results.

## Results

Between February 2021 and July 2021 nine out of 14 potential participants were included in the interview study. Three caregivers refused to participate for reasons such as emotional strain or lack of time. Two were not invited into the study because they were not proficient in the German or Italian language, respectively.

### Description of Study Participants

We recruited six participants in Germany and three participants in Italy. The sample presented here was homogenous in terms of demographic characteristics. Six of the participants were female, three of them male. The mean age was 49 years (range 26 to 75). The participants were spouses (5), children (2), parents (1) and siblings (1) of the patients. Five of the caregivers were the patient’s legal guardian or held the power of attorney. Five had a university degree and four had visited secondary school. One of the caregivers was a health professional. Two participants were related to the same patient.

Five of the DoC patients were males, and three of them females. The patients´ mean age was 56 years (range 33 to 77). Six of the patients had a MCS diagnosis and two a UWS. Mean length of interview was 46 minutes (range 34 to 70). The mean time between the medical conversations on the findings of the functional neurodiagnostic tests and the interviews was in average 21 days (range 3 to 67). The average time between the injury date and the interview date was 22 (range 11 to 34) weeks. The study participants´ as well as the patients´ descriptive characteristics are displayed in Table 2.

**Table 2:** Description of the study sample

Interview	C1	C2	C3	C4	C5	C6	C7	C8	C9
**Attributes of the caregiver**									
Age	53	59	41	61	26	38	36	75	54
Gender	female	female	female	female	female	male	male	male	female
Relationship to the patient	mother	wife	wife	wife	half-sister	son	son	husband	wife
Educational background	middle school	university degree	middle school	middle school	university degree	university degree	university degree	university degree	middle school
Country of data acquisition	Germany	Germany	Germany	Germany	Italy	Italy	Italy	Germany	Germany
Health Professional	no	no	no	no	no	yes	no	no	no
**Attributes of the patient**									
Age	33	72	41	61	37	62	62^b^	77	66
Gender	male	male	male	male	female	female	female ^b^	female	male
Cause of brain injury	TBI	HIE	HIE	HIE	TBI	Vascular	Vascular ^b^	Aspiration pneumonia	Anoxia
Duration of DoC (weeks)	33	21	12	26	18	31	34	20	11
Clinical diagnosis based on CRS-R	MCS-	MCS-	Coma	UWS	UWS	UWS	UWS ^b^	MCS-	UWS
Best CRS-R score	7	9	4	4	6	8	8 ^b^	8	5
HD-EEG Result	MCS	MCS	MCS	MCS	UWS	MCS	MCS ^b^	MCS	UWS

^b^same patient for C6 and C7

TBI = Traumatic Brain Injury, HIE = Hypoxic Ischemic Encephalopathy, MCS = Minimally Conscious State, UWS = Unresponsive Wakefulness State, CRS-R = Coma Recovery Scale - Revised

### Themes and Subthemes

**Fig. 1:**
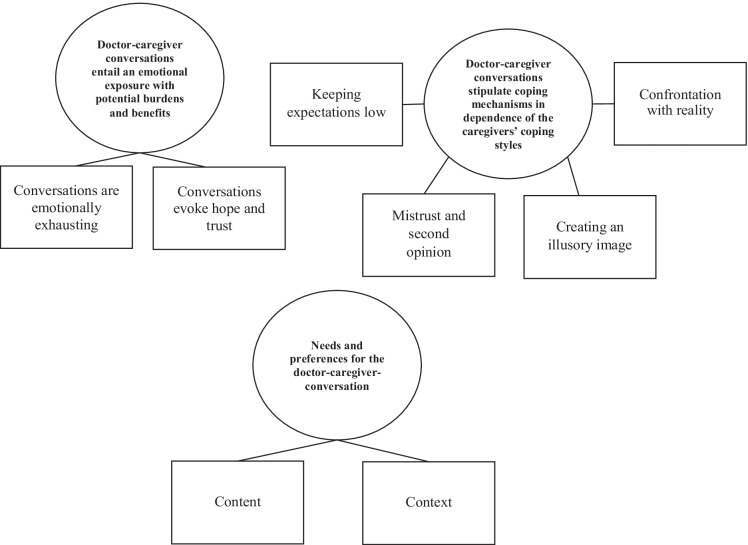
Thematic Map with three themes (ellipses) and eight subthemes (rectangles)

**Fig. 2:**
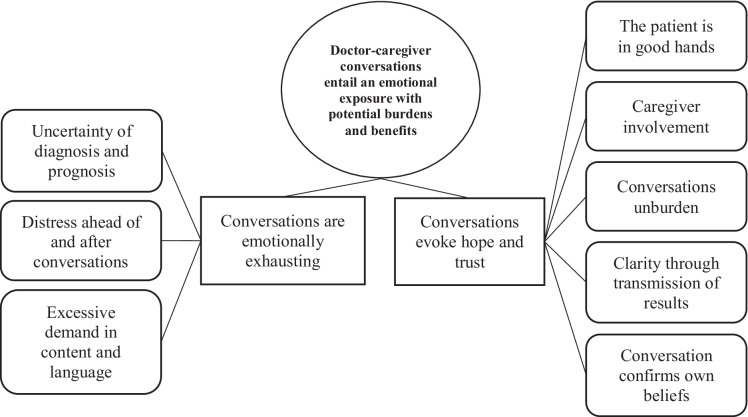
Theme 1 “ Doctor-caregiver conversations entail an emotional exposure with potential burdens and benefits” with two subthemes (rectangles) and eight categories (rounded rectangles)

**Fig. 3:**
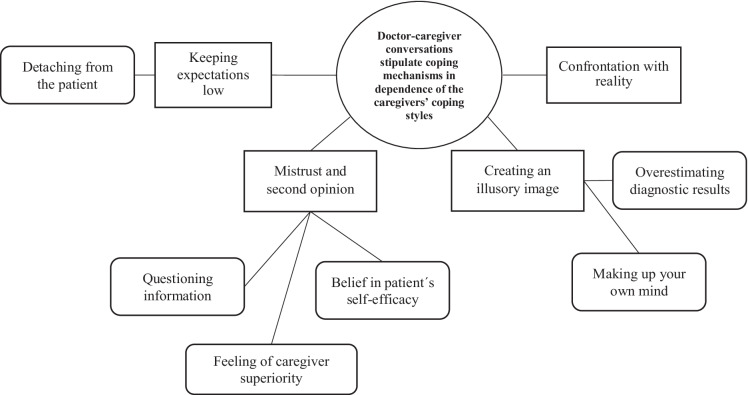
Theme 2 “Doctor-caregiver conversations stipulate coping mechanisms in dependence of the caregivers’ coping styles” with four subthemes (rectangles) and six categories (rounded rectangles)

**Fig. 4:**
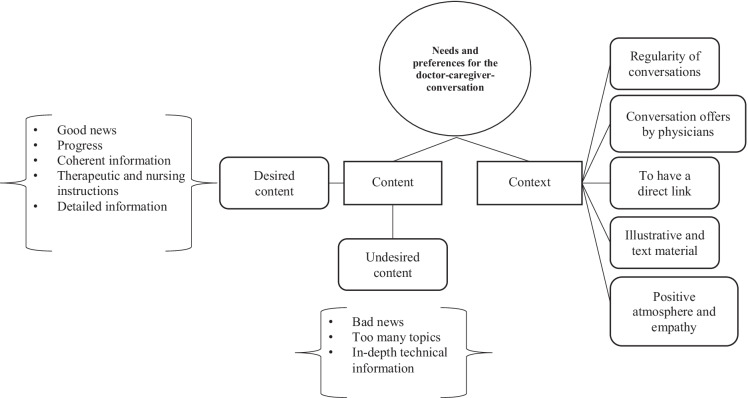
Theme 3 “Needs and preferences for the doctor-caregiver-conversation” with two subthemes (rectangles) and seven categories (rounded rectangles)

Based on the interviews data, we defined three themes, eight subthemes, 22 categories, and 345 codes. All themes, subthemes, categories and codes are shown in figs. 1,2,3,4. The themes 1 and 2 show how communication is experienced and managed, and theme 3 gives information about the participants´ needs.

#### Theme “Doctor-caregiver conversations entail an emotional exposure with potential burdens and benefits”

All participants reported caregiver-physician communication to be an emotional experience, that was often uncomfortable and stressful, while at the same time having the potential to strengthen the participants´ hopes and beliefs.

Conversations were perceived as emotionally exhausting. Participants reported distress and emotional strain ahead of and after conversations, because of the fear to receive disheartening information. Accordingly, participants stated that conversations about neurological findings caused distress and created inner unrest, turmoil and negative emotions. Consequences such as anxiety, sleeplessness and panic attacks were described.

„The three of us [the two children and spouse of the patient] are all nervy and on edge, so it’s not easy to bear one another’s games; […] Generally speaking, this thing causes some mess, or better, I don’t know how to explain it, some agitation and nervousness (3) and also anger sometimes. ” (C7, Pos. 48)

Participants felt that they were overwhelmed by the demand in content and language. The quantity and quality of the information they were confronted with were perceived as additional burden, even in those with a professional proximity to the medical field. Participants contrasted conversational styles of physicians to that of therapeutic and nursing staff, among others stating the preference of a low threshold language over medical terminology. They felt that lay language conveyed warmth while technical language was perceived as cold and discouraging.

“And then I just think to myself, okay, with [the patient] it was pretty severe, that's what I was told over and over again. I've heard that enough (laughs) and every time I talked to a doctor, he always said "yes, that's been very intense" and that's another slap in the face. That doesn’t necessarily motivate you then. That's why I had this restraint, that I now needed some space and not call a doctor, who can't tell me anything else anyway. It all takes much longer anyway. And then you automatically turn to therapists or to people who really approach it with a motivation, so that (laughs) is so soothing for your soul.” (C2, Pos. 122)

The uncertainty of diagnosis and prognosis was perceived as an additional burden as participants aimed for a reliable diagnosis and prognosis to be able to participate in the decision-making and to cope with the situation.

“Hm (sighs), yes. Well, of course you would like to know where the journey is going. But nobody can say that, you don’t know. “(C1, Pos. 59)

Participants also perceived conversations with physicians in positive ways, namely as situations that were able to seek relieve and to unburden themselves. The recognition of the emotional involvement of the participant and the empathetic willingness to meet the participant’s needs evoked hope and trust. This was especially the case, when the information transmitted had a positive value for them because it was in line with what they hoped for. Participants experienced this as a relieving event that strengthened and supported their hope for and belief in recovery.

„Um (3). So, before the conversation with [the neurologist], I also panicked insanely, and had sleepless nights, too. But the conversation was very positive for me because I was told that certain functions were still in place or that the nerve network was still in good shape. And that my loved one shows minimal consciousness. “(C1, Pos. 29)

The allocation of large parts of the physician’s time and effort was perceived as an indication of care and security. When physicians invested resources in the conversations with them, participants expressed their confidence in the facility and that the patient and even they themselves felt like they were in good hands. Participants felt like they were taken seriously with the time and affection dedicated to them.

„On the contrary, in rehabilitation I have always had the feeling that there were however changes, that there were improvements and that important attentions were given to my sister and also to the family member. So, from the time I had the conversation with the doctor, for me it was just like a confirmation of the fact that I was happy about both where she was and the therapeutic path she was following. But also from the point of view that for me it was simply important that the neurologist who cares for my sister took 40 minutes of her time for the radiograms, the exams and so on. (3) […] I live far away, so I can be a sort of additional weight, an additional obligation, which was instead dealt with. (3) I don’t know, I felt like something the doctor also wanted to do, not like a job, an additional weight that the doctor had to take care of at the end of the day, quite the opposite.” (C5, Pos. 47)

Moreover, participants perceived conversations as situations that created clarity and safety, underlining their need to receive a clear picture of the patient’s condition. These conversations helped them grasp the serenity of the situation and to accept it. They were described to be essential to participants as they were a tool to understand and a key to the interpretation of the ilness.

“Um, the fact that she managed to find some time and devoted to us some room to explain, in the most clear and comprehensible way as possible, the data. Um, the clinical data she gathered, for us, this was not banal at all, on the contrary. Concerning details, the thing I remember best is the clinical conditions, so the fact that she told us about functions, explained to us what kind of exams she performed. […] Well, I remember that this exam was useful to understand the real clinical conditions of my mom. And then, I remember very well that she talked about different areas, dividing them into the motor area, the cognitive one, the visual one and the auditory one, explaining to us that there were areas which were more impaired and other areas which were less impaired. “(C6 Pos. 15)

#### Theme “Doctor-caregiver conversations stipulate coping mechanisms in dependence of the caregivers’ coping styles”

Participants understood information disclosed in the caregiver-physician communication as an invitation to confront themselves with reality in all its complexity yet it was accompanied by emotional pain to fathom and understand their consequences. The information provided was considered a key to learn, accept, and deal with the reality as it presents itself. It was preferred over not knowing and dealing with uncertainty.

“Absolutely. For me, knowing is important, because if I know, I can then handle it on my own, handle what is happening. […] I prefer to receive information constantly, probably that’s part of my character, the continuous search for information is important for me in order to stay quiet. Of course, some people prefer not to know; instead, for me, it is necessary to know in order to understand and then to process information and, as a consequence. I realize that it’s a condition that makes me feel better. Overall, the information is clear and is very useful to me. I noticed that not having information causes me even more turmoil at the emotional level; also with my other relatives, it becomes far more complicated.” (C7, Pos. 44)

Participants were carefully managing their expectations based on experiences prior to these conversations. Keeping the expectations low and being pessimistic about new information prevented them from suffering setbacks and disappointments. In some participants, this was a reaction to previous conversations with medical staff.

„No, I actually didn't have any [expectations]. We once asked what the current condition was, neurologically, um (3). Well, I spoke to the neurologists in the hospital in [location of previous facility], they showed me the picture of his brain and what was damaged there. My expectation was (3), well, I had no expectations. They then told me what was actually in front [the frontal brain], what was going to break, and what he originally/ So, in the hospital there in [location of previous facility], they said that he had no chance of ever getting better. Well, I would have to expect the worst. And then you don't have a lot of expectations anymore (laughs). Therefore what happened here, is a miracle. “(C2, Pos. 32)

Another way of the participant’s coping was to build some sort of illusory image that was in line with their hopes and beliefs. Participants expressed that they preferred seeing the patient with their own eyes beyond the information provided within the caregiver-physician conversations as it helped them form their own picture of their relative’s condition. This picture tended to be characterized by exaggerated hope for the patient’s recovery and was a way to possibly defuse the harshness of the situation. One participant expressed very high hopes in and overestimated functional neurodiagnostics to be a steppingstone for further treatment whereas it also had the potential to terminating rehabilitative treatments.

“That he, so, that [the patient] can benefit from having a more accurate picture, so that you can then be more specific about the skills that he then has left. So that [the patient] is sort of standing on the springboard and help him to make the leap to learn certain skills.” (C2, Pos. 86)

Furthermore, participants reported to cope with the situation by mistrusting and disregarding information that was being provided by physicians. Turning to other physicians or medical staff in different medical facilities for secondary opinion seemed to be essential to them so that they could choose between different professional opinions. What they usually chose to believe was information that was positive from their point of view and affirming of an ongoing medical treatment. Mistrust and defiance seemed to be shaped by the participants´ belief in the self-efficacy of their loved ones and their capability to take a stake in their own recovery process as well as the participants´ feeling of superiority towards physicians. The way their loved ones had dealt with difficult situations in life before was chosen as the maxim for the current situation.

“Yes, well I do know my son, I know that my son is fighting, that he is a fighter. He always has been and yes, that's, yes, I'll say it like this, you cannot bury your head in the sand. Because that doesn't help my loved one and that doesn't help me either. And that's why I believe in him, I still have hope. I see that he reacts and yes, that's how I keep my head above water, I say.” (C1, Pos. 67)

#### Theme “Needs and preferences for the doctor-caregiver-conversation”

Based on their experiences, participants expressed needs and preferences for doctor-caregiver conversations as such. Here we developed two subcategories for textual needs as in content which is (1) information that they want to receive and (2) information they don’t want to receive. Participants stated that they looked for positive as in optimistic information and focused on that information within conversations. Negative reports were discarded by participants, because they didn’t want to be confronted with them or refused to believe them. Positive information is necessary for participants to support their coping with the situation and to stabilize their hopes and beliefs.

“Yes, yes, so of course one is afraid of, you know, that one, well, you refuse to believe that. You refuse to believe it, you only want to hear the good. And everything that is bad, for example, I know that he is blind on the right eye. You do struggle with that (seems very emotional).” (C1, Pos. 127)

Similarly, participants expected information that indicated progress and did not want to hear about stagnation in the rehabilitation progress. Participants seemed to have high expectations of diagnostic and therapeutic procedures and thus expected the patient to recuperate automatically in the course of time.

“Well yes, you would like to hear that there is an improvement, that there are small prospects for a change. I think everyone would feel that way when a relative is in rehab. And with rehab, in my eyes, the goal is simply that the person or his quality of life improves again through rehab.” (C4, Pos. 47)

Participants also expressed the need to receive coherent information. Incoherence was perceived when diagnostic test results contradicted each other or when caregivers heard differing secondary opinion by other physicians. Mostly this need for coherence seemed to arise from the difficulty to diagnose consciousness accurately and definitely. Participants expressed the need for certainty and clarity so that they could start accepting their loved-ones´ condition.

“Hm (affirmative). And with the other doctors I only spoke on the phone, and that was always just a two-minute conversation and everyone said something different, and, yes.” (C3, Pos. 145)

In one of the interviews, in order to prepare for domestic care the need to receive instructions on therapeutic and nursing measures was expressed. The participant signaled optimism for the patient’s recovery and the desire to participate actively in the therapeutic process. Information on how to actively participate in the care and treatment seemed to empower participants and give them back a sense of control as well as a deeper understanding of their loved-one’s condition.

“Yes, just, how you can support him, no matter what, at any time. And when you see him then, and he's just babbling something (laughs)/ [what I need to know is] how you can win his attention and or what you can do, or what you can give him, so that/. He was also very, he had a really cramped hand at the beginning and I didn't know that it would loosen up later on. And afterwards, I read one specific book and then I saw, those are probably such phases that they go through. I found that very interesting with the book. I think it's from here, or/ Anyway, we got ourselves two books. That’s what it said.” (C2, Pos. 138)

Detailed information on the current diagnosis, the prognosis and the classification of the severity of the condition seemed to be the most important informational need of participants. Participants stated the need for information to be clear and detailed in the sense of giving justifications for main causes of certain developments. Receiving directions on how the condition may evolve in the future was essential to participants as they were to decide on further treatment options.

“I was driven by curiosity, by a will to know, an interest. I was not distressed in the least. I mean, considering my experiences, there is grief about the tragedy that happened but not about the conversation itself, instead I was happy to be able to talk with the doctor, for the opportunity to understand more, to give meaning to the plates [the MRI scans], to feel closer to my sister.” (C5, Pos. 21)

Confrontation with pessimistic perspectives of the patient’s prognosis, especially end-of-life scenarios, or suggestions to change the therapeutic goal and consequently not escalate or terminate life-sustaining treatment, was not appreciated by the participants in this study. This seemed to be not only due to the emotional strain associated with it but also with the great responsibility participants perceived as surrogate decision-makers. When asked about negative conversation experiences with doctors outside of the study site, one participant described the following:

“So the conversations that I had with the doctors before [at the previous hospital], they were, it was just about the fact that if my son gets an infection or something at some point, that I should stop all life-sustaining measures, I should sign for that. And it was *only* about that in those conversations, it was about my son not having a chance anymore, it was about him staying like that for the rest of his life. That he will no longer be able to experience anything, that he is practically brain dead. […] So it was never about what was done, which therapies my son receives. Rather, it was really only about "Keep in mind that nothing more can be done for your son. He doesn't feel anything anymore, he doesn't notice anything anymore". Um, yes, just that I should then take off these life-sustaining measures, if he has pneumonia or something, that he is no longer connected to devices, that I should then just let him die.” (C1, Pos. 45-51)

It was further mentioned that focusing on one specific topic during one appointment instead of addressing multiple issues helped the participants gain a better understanding for the diverse and complex topics associated with the treatment of their loved-one.

“But well, for example also when we came here to the facility together it was a bit more dispersive, maybe because of the great amount of topics […] It was at [the patient]´s admission maybe, and she had had more exams, maybe less specific, but I remember that we had a similar conversation. It was much more of help to me that we selected only one topic” (C5, Pos. 17)

However, participants did not express the need for in-depth technical information on the functional neurodiagnostic procedures, but rather developing a general idea of its meaning and the consequences of such as they were important for them to develop an understanding.

“Being ignorant, so to say, I was interested, because you anyway get to know technical, specific things that, obviously, I would have preferred not to know. But, well, anyway, they contribute to one’s knowledge and […] I’ve become very much knowledgeable about blood tests, about hemoglobin; […] After all, it’s about my mother.” (C7, Pos. 36)

Another field of interest were contextual factors that participants reported within the caregiver-physician conversation. They expressed the wish for medical information to be delivered in a positive and hope inducing way rather than in a factual manner. While some of the participants were satisfied with a very clear conveyance of results, others perceived it as cold and disheartening. Yet, all participants stated to prefer a conversational style that was calm and empathetic, generating a positive atmosphere. *How* information was conveyed played a major role in how participants perceived conversations and how they managed the obtained information. A motivational conversational mentality that was often presented by therapists seemed to empower participants more where a reportedly depressing conversational mentality that was rather related to physicians seemed to discourage them. This led them to listen selectively to sources of information.

“So, for me it was a situation in which, um, then at some point I decided, I actually no longer wanted to talk to the doctors, that hit me too much. So, it hits me too, um, emotionally. Because the doctors are just very, very professional, factual and put the facts on the table and for me, that (2), yes, how do you say? (*incomprehensible*) There is no alternative (laughs). Fact. And then, when you talked to the therapists, they were so incredibly motivated and they said "no, [the patient] is making progress and he can stand now and he's coming to walk and we've talked to him now and he can do this." So, there's been such a positive mentality there. So, of course, I much preferred to talk to the therapists than to the doctors (laughs).” (C2, Pos. 18)

Furthermore, time aspects such as frequency of conversations and offering time to participants was understood as a display of care and support to caregivers. Spending time on the conversation about their loved ones helped participants feel close to them, be emotionally involved in their care and helped them to understand the patient’s condition and development.

“No, just think that we come from a place where they did not call you and it was you who had to look for information, to remember the name of the doctor you had talked with for two seconds and beg him to give you some news. […] here in [current facility] the approach is extremely different; you receive information on a continuous basis, even if it is bad, and they tell you about the situation. […] also in the conversation with the doctor we were given time to ask all the possible questions. For us, this was a very important factor, even if, like the last times, news was not positive, at least you know it. (C7, Pos. 42)

Another contextual aspect that seemed important to participants was physicians approaching caregivers, proposing conversations actively. On the grounds of past unpleasing or hurtful experiences, some participants stated to have turned away from the caregiver-physician communication. Others felt like an additional workload to physicians and therefore were inhibited to request a meeting or call. These tensions have been intensified during the COVID-19 pandemic where participants had to comply with strict visitation rules to protect the vulnerable patients.

“Yes, two ideas that come to my mind to try and improve a conversation which, in my opinion, was anyway good. I mean, I wish they were all like the one we just experienced; I don’t want to bore you but in [the previous facility] we had appalling experiences, in which you had to look for the doctor and, maybe, you managed to contact him and somebody would answer after you had tried many times. This was the standard (3). You can imagine how stressful it was for the relative who is experiencing such a tragedy and receives no information.” (C6, Pos. 57)

Having a primary contact physician as a direct link between the patient and caregiver was reportedly helpful for participants, as they felt invited to build a relationship that allowed them to ask and share all their upcoming questions and doubts.

“Not at all. Actually, everything was great. If I may say this, we have now created a bond with you [the medical staff], there is something that probably connects us directly with mom, so for us, talking with you, being in relation with you is fundamental to be connected to her. Actually, I want to thank you and all of you for this, for what you are doing.” (C6, Pos. 76)

Participants also expressed the need for the use of visual and text material alongside the disclosure of diagnostic results. Graphics seemed to help them better understand the complexity of the situation, breaking it down from an abstract phenomenon to a concrete and clearer picture. Text material on the other hand was stated to enable them to prepare for conversations in advance or to reiterate, consolidate and share with others what had been said. What they seemed to essentially look for, was a comprehensible and concise summary of the patient’s condition.

“At the time when I was there, new results would have been open, but they were not processed and it was also said that I would receive a message. And at that time I asked for, or I asked whether it would be possible to make a copy of the pictures and graphics. […] And I looked for a short summary, because I said I would like to clarify at home now, because for [the neurologist] it was easy, he had the pictures available and explained to me on the basis of the pictures. And then I just said, I'm going home now and I can't / it's just more difficult without having something in my hand to explain it at home. I then tried it that way, I hope that I have reproduced everything correctly. So, I think that my children have already understood it the way I have understood it here. […] So, I was just promised that I would get it, short and to the point, so that you can explain it again at home in an understandable way.” (C4, Pos. 29)

## Discussion

This is one of the first studies that explored the perceptions and needs of caregivers in the context of doctor-caregiver conversations about the technology-enhanced diagnosis and prognosis of the DoC patients´ current and future conditions in acute rehabilitation. The results have the potential to inform physicians how their participation in these conversations is perceived by an important and so far neglected group, and guide their efforts for effective communication of results of complex neurodiagnostics measures.

In this analysis of qualitative interview data, we have described that caregiver-physician conversations strongly expose caregivers emotionally, which is perceived positively and negatively by them. Caregivers cope with the obtained information in different ways, namely by keeping their expectations low, mistrusting, and looking for secondary opinion, creating an illusory image or by confronting themselves with reality. Our results suggest informational needs that are grounded in the coping process with the loss of a loved one despite his ongoing need for care and support, namely the need for good news, information on progress, coherent and detailed information, and information regarding therapeutic and nursing instructions. Moreover, the regularity of conversations and information, invitations for conversation initiated by physicians, having a direct link to the treatment team, the use of illustrative and text material as well as positive atmosphere and empathy are contextual needs that we identified within the analysis.

Our findings indicate that severe stress and burden is experienced by caregivers through caregiver-physician communication due to the uncertainty and ambiguity of the situation and the responsibility to make informed decisions that reflects the recipient’s will. This is in good agreement with previous research reporting high emotional burden in DoC caregivers [8, 24, 37, 38]. Yet we were also able identify beneficial aspects of caregiver-physician communication, pointing out the importance of the comprehensive conveyance of the meaning of diagnostic test results bringing about a feeling of safety, understanding and closeness to the patient.

Our findings are consistent with previous research of Schembs et al. (2020), stating that the information on functional neurodiagnostics are perceived as an attack on caregivers´ belief systems [39]. Whereas favorable information stabilized caregivers´ belief systems, unfavorable or negative test results destabilized their belief system. In order to uphold hope and positive feelings, caregivers selected information, turned away from medical staff or restabilized their belief system by “insisting to be the better expert for the patient’s evaluation” [39]. However, it is necessary to point out that all participants presented in Schembs et al.´s work are characterized by high hopes for recovery in their loved-ones and thus are not entirely comparable to our sample. Nevertheless, a similar observation within our analysis, where caregivers highlight positive information and question negative information, or a reality that is unacceptable to them, has been made previously [39, 40]. Paired with the considerable uncertainty of a correct assignment of diagnosis and prognosis, this phenomenon is critical to the shared decision-making process, where physicians and caregivers mutually participate. It highlights a mismatch between the professional perspective and the lay perspective of caregivers on scientific results: while professionals try to determine the condition of the patient with regard to reproducible signs of consciousness, caregivers might adopt a biased reasoning process that could negatively influence the decision-making process about the appropriate health care strategy. In ethical guidelines for end-of-life decision-making for patients with DoC this possibility should be adequately acknowledged. In clinical practice, clinicians should be aware of this possibility and they should try to reduce its impact on decision-making by adequate provision of scientific information.

In-depth technical information of diagnostic procedures took a toll on caregivers, who yet expressed the need for detailed information and to receive an overall view over the patient’s condition and its consequences. Even if based on facts, negative information was not appreciated by caregivers in this study and lead to turning away from the caregiver-physician communication. This observation is in line with the hypothesis that caregivers rather show the need for support instead of a need for actual information [41]. Yet it poses a challenge to the newest demands to a full and transparent disclosure of diagnostic results [42, 43]. Nevertheless, our findings may contribute to postulations in literature, namely to explore the impact of disclosure of diagnostic test results and to develop educational material for DoC caregivers [44].

With our qualitative study we aimed to gain an understanding and give a voice to caregivers of patients with disorders of consciousness to express their experiences and needs regarding caregiver-physician conversations for the disclosure of functional neurodiagnostic test results. However, our interview study is limited considering linguistic inclusion criteria, mandating a fluent language level in German and Italian to be able to participate. Potential participants with different mother tongues should be communicated with and included within care research. The authors of this study have backgrounds in neuromedicine, (clinical) psychology, ethics, nursing and epidemiology. Throughout the study, we were aware of the fact that our respective backgrounds and previous understandings might influence the research process. Especially in the case of one interviewer, who also did psychological counselling with caregivers, the relationship that had been created before the study may have influenced the information provided within the interviews. The nursing background of the first author responsible for the analysis might as well have influenced the interpretation of data. On the other hand, the multiple backgrounds of our authors may be a strength in our study as we met several times to discuss the study design, preliminary results and share our understandings of data.

Overall, we strove to openly interpret the data and transparently report our research process from the research design to the results. The vulnerable position of the participating caregivers may have had an influence on the quality of interviews. Yet, the psychological backgrounds of our interviewers enabled them to react adequately and empathetically to the participants´ emotional states and ease the interview situation, which may be considered a strength of the study.

The mandatory use of facemasks during the in-presence interviews as a COVID-19 measure may have inhibited the emergence of a relationship between the interviewer and the participant as well as openness in the conversation. As some of the interviews took place in the examination room of the neurologist who had disclosed the results of functional neurodiagnostic testing, it is possible that the information provided was influenced by the interview setting. A more neutral place may have helped the participants to convey their viewpoints more openly.

The different cultural backgrounds of the sample might as well influence the results of this study. Despite a lacking possibility to generalize these study results, our results may be applied to similar situations or individuals.

## Conclusion

The crucial role and the important responsibilities of informal caregivers in patient care have gained their legitimate attention in clinical research and practice. Caregivers of patients with a DoC are confronted with surrogate decision-making while experiencing a life changing and burdening situation. The emotional involvement of informal caregivers requires a particularly sensitive handling on the one hand and an efficient communication strategy on the other hand.

Our results show that caregivers might be prone to biased reasoning selecting information according to their belief system, yet they want detailed information to gain a deep understanding and a clear picture of their loved-ones´ condition. Balancing these somewhat contradicting needs surely is a difficult task in clinical practice, which needs to be addressed in further research.

We strongly suggest further development and a more comprehensive use, reporting and communication of functional neurodiagnostics. The determination of recovery potential and patient survival depends among others on safe diagnostic pathways. Our findings also point out the importance of effective as well as comprehensive communication and future efforts for the prevention of long-term mental health issues in DoC caregivers is required. Yet, the understandable need of caregivers to receive precise diagnostic and prognostic information may be unachievable, given the complex nature of severe brain injury and the lack in sensitivity/specifity of functional neurodiagnostic testing.

Future efforts should aim towards empowering DoC caregivers by acknowledging their role as surrogate decision makers and to meet their emotional as well as informational needs. The doctor-surrogate information and conversation is key to building trust in the care facility. We suggest implementing elaborate information interventions in the form of educational material such as brochures for caregivers with both illustrative and text material, translating the meaning of neurodiagnostic test results with regard to their importance for clinical practice. Such structured interventions should encompass precise, short and comprehensible information for laypeople on the diagnosis and prognosis of disorders of consciousness as well as therapeutic options and consequences. They should be complemented individually with information on the specifics of the caregivers´ respective loved ones. Moreover, contact information on mental health professionals or support groups may be relevant to care for caregivers. A brochure as such can additionally serve as a guideline for physicians to structure a conversation, in which diagnostic test results are being conveyed and may help to communicate more effectively if the caregiver is in need of such background information. Moreover, training for health professionals may be a powerful tool to improve and ensure an empathetic and effective communication of results (under uncertainty) and create awareness for complexities the vulnerability of caregivers within that professional group. Assigning direct links to DoC caregivers and offering them comprehensive conversations regularly should furthermore be implemented within the diagnostic pathway.

## Supplementary Information

ESM 1 (PDF 403 kb)

## Abbreviations

CMD: Cognitive Motor Dissociation
DoC: Disorder of Consciousness
MCS: Minimally Conscious State
TA: Thematic Analysis
UWS: Unresponsive Wakefulness Syndrome
