# Supplementary material for: Informal Caregivers of Patients with Disorders of Consciousness: a Qualitative Study of Communication Experiences and Information Needs with Physicians
Source: Neuroethics. 2022 Jul 23;15(3):24. doi: 10.1007/s12152-022-09503-0 (PMC9307713; doi:10.1007/s12152-022-09503-0)
Supplement: Supplementary file 1 — (PDF 403 kb) [file 12152_2022_9503_MOESM1_ESM.pdf]

PerBrain Qualitative Study – Semi-structured Interview Grid

Nr.	Leading question (invitation to tell)	Check: was the following element mentioned?	Concrete questions	Questions to continue and guiding questions
(1)	We are interested in your experience with the last counselling (the last big conversation that you had with the doctor/medical team) where you were informed about the neurological examinations of X[1]. Please, tell me all you think is important in this regard. Simply start from what comes to your mind first.	• Experience (a story)<br>• Facts:<br>- Participants (doctor/nurse/psychologist/others?)<br>- Content (tests/test results)<br>• Emotions: during/after	A) What happened during that appointment?<br>B) What happened since then?	• Can you tell me more on the topic?<br>• Can you make an example?<br>• And then? How did it go on?<br>• And what happened with…?
(2)	In how far did that conversation differ from other experiences of medical communication?	• Experiences (stories, personal)<br>• Relevance/meaning		
(3)	What have you expected of the neurological examinations that were conducted here?	• Needs for information<br>- Kinds of information<br>- Delivery<br>- More/less	A) What specific kinds of information from the medical team are important to you at the moment?<br>B) How would you like that information to be delivered to you?<br>C) What kinds of information do you need more/less of?	

[1] Name or role of the family member

Nr.	Leading question (invitation to tell)	Check: was the following element mentioned?	Concrete questions	Questions to continue and guiding questions
(4)	Could you tell me more about X's specific neurological examinations?	• Procedures<br>• Neurodiagnostic methods:<br>- High-resolution EEG<br>- Functional MRS<br>- TMS/EEG<br>- PET<br>• Results<br>- Level of consciousness of the patient<br>• Diagnostic category	A) What different kinds of examinations are you aware of?<br>B) How would you summarize these examinations in your own words?	• Can you tell me more on the topic?<br>• Can you make an example?<br>• And then? How did it go on?<br>• And what happened with…?
(5)	How were the results of these neurological examinations communicated to you?	• Context of delivery<br>- Sufficient time<br>- Competent and understanding<br>- Quiet or hectic environment<br>- Shown images?<br>- Opportunity to ask questions<br>- Specialist jargon vs simple language<br>- Simplicity/complexity	A) How did you experience the transmission of these results?	

Nr.	Leading question (invitation to tell)	Check: was the following element mentioned?	Concrete questions	Questions to continue and guiding questions
(6)	Could you describe how that affected how you think about the patient?	• Emotions: fear, worries/hope, confusion, despair, more unclear than clear<br><br>• Understanding of illness:<br>- Patient is responsive/unresponsive; aware/unaware, able to feel pain/not able to feel pain, able to understand/not able to understand;<br><br>• Treatment strategy:<br>- more effort in rehabilitation<br>- more treatment for pain<br>- “wait and see”<br>• Limitations of life-sustaining treatment and palliative care	A) How was your immediate reaction to the result communication?<br>B) What feelings did they evoke?<br>C) What do you know now that you have not known before?<br>D) How did the communication of the results of these exams influence your understanding of the condition /and possible future of X?<br>E) How did it influence, what you consider to be the appropriate treatment strategy for your loved one?	• Can you tell me more on the topic?<br>• Can you make an example?<br>• And then? How did it go on?<br>• And what happened with…?

Nr.	Leading question (invitation to tell)	Check: was the following element mentioned?	Concrete questions	Questions to continue and guiding questions
(7)	In how far could the delivery of neurological information about X be improved in your point of view?	• Evaluative part: Critic/Room for improvement:<br>- Illustration material<br>- More basic information on the functioning of the brain<br>• Easier language	A) What worked well for you and what would you wish to be changed in future conversations like these?<br>B) Was there something missing that you could have benefitted from?	• Can you tell me more on the topic?<br>• Can you make an example?<br>• And then? How did it go on?<br>• And what happened with…?

(8) Is there anything that is important to you, that we have not talked about enough during our interview?
